# Maslinic Acid, a Natural Triterpene, Induces a Death Receptor-Mediated Apoptotic Mechanism in Caco-2 p53-Deficient Colon Adenocarcinoma Cells

**DOI:** 10.1371/journal.pone.0146178

**Published:** 2016-01-11

**Authors:** Fernando J. Reyes-Zurita, Eva E. Rufino-Palomares, Leticia García-Salguero, Juan Peragón, Pedro P. Medina, Andrés Parra, Marta Cascante, José A. Lupiáñez

**Affiliations:** 1 Department of Biochemistry and Molecular Biology I, Faculty of Sciences, University of Granada, 18071, Granada, Spain; 2 Department of Experimental Biology, Biochemistry and Molecular Biology Section. University of Jaen, 23071, Jaén, Spain; 3 Department of Organic Chemistry, Section of Natural Products, Faculty of Sciences, University of Granada, 18071, Granada, Spain; 4 Department of Biochemistry and Molecular Biology, Faculty of Biology, University of Barcelona, 08028, Barcelona, Spain; Universidad Pablo de Olavide, Centro Andaluz de Biología del Desarrollo-CSIC, SPAIN

## Abstract

Maslinic acid (MA) is a natural triterpene present in high concentrations in the waxy skin of olives. We have previously reported that MA induces apoptotic cell death via the mitochondrial apoptotic pathway in HT29 colon cancer cells. Here, we show that MA induces apoptosis in Caco-2 colon cancer cells via the extrinsic apoptotic pathway in a dose-dependent manner. MA triggered a series of effects associated with apoptosis, including the cleavage of caspases -8 and -3, and increased the levels of t-Bid within a few hours of its addition to the culture medium. MA had no effect on the expression of the Bax protein, release of cytochrome-c or on the mitochondrial membrane potential. This suggests that MA triggered the extrinsic apoptotic pathway in this cell type, as opposed to the intrinsic pathway found in the HT29 colon-cancer cell line. Our results suggest that the apoptotic mechanism induced in Caco-2 may be different from that found in HT29 colon-cancer cells, and that in Caco-2 cells MA seems to work independently of p53. Natural antitumoral agents capable of activating both the extrinsic and intrinsic apoptotic pathways could be of great use in treating colon-cancer of whatever origin.

## Introduction

Several nutraceutical properties have been attributed to different triterpenes, in general, and to maslinic acid (MA) in particular, whose antitumoral effects have been extensively evaluated in different human adenocarcinomas. Colon cancer is the second leading cause of cancer death in humans after lung cancers. Hence, we focus here on the apoptotic mechanisms triggered by MA in Caco-2 colon-cancer cells, which are deficient in p53 protein. Two major pathways have been described in the apoptosis induction mechanism: the extrinsic or the death-receptor pathway and the intrinsic or the mitochondrial pathway. The extrinsic pathway is normally defined by caspase-8 activation. This cysteinyl-aspartate protease is recruited by the adapter molecule FADD, which is associated with the death domain of death receptors such as FAS, TNF-R1 or TRAIL, upon ligand binding [1–3].

Active caspase-8 has been shown to cleave directly and activate the caspase-3 protease effector, which in turn activates other substrates either directly or indirectly to finally induce apoptosis. The intrinsic apoptotic pathway, on the other hand, is associated with the activation of proteins such as Bax that belongs to the Bcl-2 family. These proteins cause mitochondrial disruption and the release of pro-apoptotic mitochondrial factors such as cytochrome-c, which interacts with Apaf-1 and activates caspase-9, which in turn proteolytically activates caspase-3 down-stream [4,5]. Finally, the activation of caspase-8 through the engagement of the death receptor can also trigger the mitochondrial pathway via Bid, a pro-apoptotic member of the Bcl-2 family. This activation of the mitochondrial pathway is believed to amplify death-receptor-induced apoptosis [6].

There has been growing interest in the use of plants as a potent source of new therapeutic antitumoral drugs. A variety of plant secondary metabolites have been assayed as chemopreventative agents against cancer [7]. Triterpenes have been reported as being major active ingredients in traditional herbal medicine. Their different biological and nutraceutical effects have been described including anti-inflammatory, hepatoprotective, analgesic, antimicrobial, antimycotic, virostatic, immunomodulatory, and metabolic and growth effects [8–18]. Some natural triterpenoids, such as oleanolic, betulinic and ursolic acids and their synthetic derivates,2-cyano-3,12-dioxoolean-1,9-dien-28-oic acid (CDDO), the methyl ester, CDDO-Me, and imidazolide, CDDO-Im, have been shown to exert substantial antitumor effects.

The induction of the extrinsic apoptotic pathway has been described in response to many of these compounds involved in caspase-8 activation. The activation of caspase-8 has been reported in apoptosis induced by betulinic acid in brain-tumour cells [19].Induction of apoptosis by CDDO or CDDO-Im has been described as being mediated by the activation of DR4, DR5 and caspase-8 [20,21]. An isomeric mixture of 3-alpha 24-dihydroxyurs-12-ene and 3-alpha 24-dihydroxyolean-12-ene, up-regulates the expression of cell-death receptors DR4 and TNF-R1, leading to caspase-8 activation [22].

Amooranin-AMR (25-hydroxy-3-oxoolean-12-en-28-oic acid) induces extrinsic apoptosis in p53-independent breast-cancer cells without affecting Bax levels in MCF-7 cells [23].Other triterpenoids such as acetyl-keto-beta-boswellic acid (AKBA) have been found to cause apoptosis via caspase-8 and DR5 activation [24]. Lupeol induces FAS-dependent apoptosis through the activation of FADD and caspase-8 [25], whilst ginsenoside Rk1 does so through the activation of caspases-8 and -3 [26], and the cucurbitaceous triterpenoid DHCB (23,24-dihydrocucurbitacin B) via the activation of caspases-8 and -9, probably by death receptor activation on the cell-surface [9].

Furthermore, we found that MA is efficient against intestinal tumor development in the Apc^(Min/+)^ mice model, suggesting its chemopreventative potential against colorectal cancer [27]. We have previously described that the intrinsic apoptotic pathway is triggered in HT29 cells in response to MA [28–30]. This difference in the activated pathways may be related to differences in the expression of apoptotic proteins in either cell type: HT29 cells expressp53 protein whereas Caco-2 cells do not. The over-expression of p53 in HT29 cells in response to MA could explain the major cell-cycle arrest, differentiation and late caspase-3 induction. The fact that p53 gene is deleted and mutated in Caco-2 cells and no detectable corresponding protein [31,32] may well explain the lower differentiation and early caspase-3 activation observed.

We have evidence to show that MA exerts anti-proliferative and pro-apoptotic effects in Caco-2 colon-cancer cells. It also induces morphological changes that are characteristic of apoptosis, such as chromatin condensation and fragmentation, as well as cell shrinkage [33].

We report here on the activation, in a very short time, of the extrinsic apoptotic pathway in Caco-2 cells in response to treatment with MA. We describe the apoptotic changes and the percentage of apoptotic cells, as determined by flow cytometry, and propose a possible molecular mechanism to explain these processes. Our results suggest that MA acts by directly increasing levels of caspase-8 and caspase-3 whilst leaving the expression of Bax and caspase-9 unaffected. All our data suggest that MA inhibits the proliferation of Caco-2 cells by directly activating the extrinsic apoptotic pathway. Natural agents that suppress the proliferation of malignant cells by inducing apoptosis may represent a useful mechanistic approach to both the chemoprevention and chemotherapy of cancer. Thus MA, isolated from olive pomace, may provide a useful new therapeutic strategy for colon carcinoma.

## Materials and Methods

### Materials

Dulbecco`s modified Eagle`s medium (DMEM), phosphate buffered saline (PBS), 3-(4,5-dimethylthiazol-z-yl)-2,5-diphenil tetrazolium bromide (MTT) and propidium iodide (PI) were from Sigma (St. Louis, MO, USA); foetal calf serum (FCS) and penicillin/streptomycin were from Gibco-BRL (Eggenstein, Germany); annexin V-FICT were from Bender Med-Systems (Vienna, Austria); primary antibodies, rabbit polyclonal anti-caspase-3 and anti-caspase-9 were from Cell Signaling Technology (Danvers, USA); anti-caspase-8 was from BD Biosciences (Erembodegem, Belgium); anti-Bax and anti-rabbit secondary antibody were from Santa Cruz Biotechnology (Santa Cruz, California, USA); culture flasks and well-plates were from Techno Plastic Products (Trasadingen, Switzerland). All other reagents used were of analytical grade.

### Drugs

**Fig 1:**
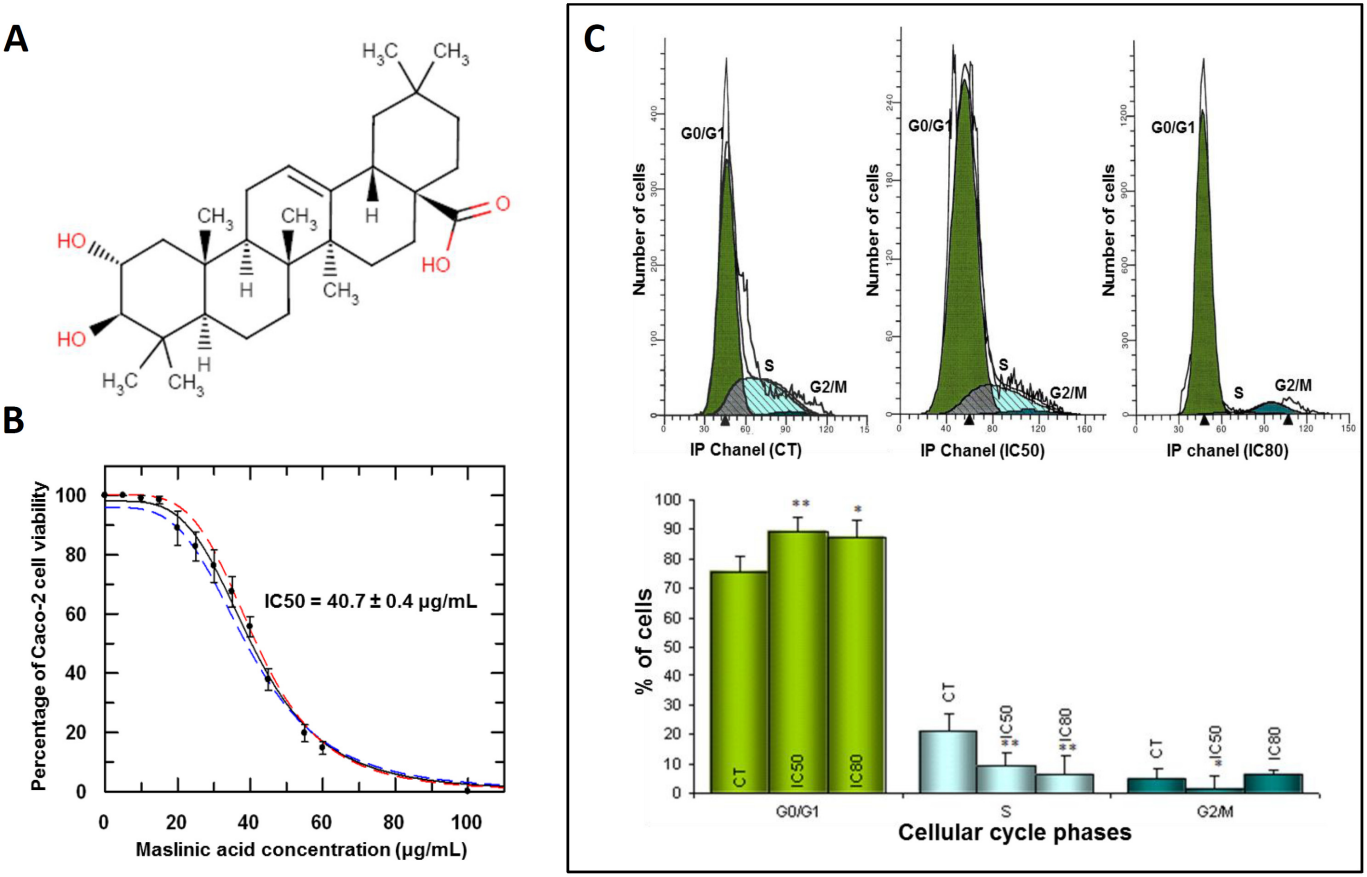
**(Panel A)** Structure of the natural pentacyclic triterpene, maslinic acid [(2α, 3β)-2,3-dihydroxyolean-12-en-28-oic acid]. **(Panel B)** Inhibitory effect of maslinic acid on the viability of Caco-2 cells. IC_50_ is the concentration of MA required for 50% growth inhibition. Each point represents the mean value ± S.D. of at least three independent experiments performed in triplicate. **(Panel C)**Top: histograms of Caco-2 cell cycle after 72 h treatment with MA. Bottom: percentage of cells in each of the cell-cycle phases. Caco-2 cells were untreated (first column) or treated with MA at IC_50_ (second column) or IC_80_ (third column) concentrations. Cell-cycle analysis was conducted after propidium iodide staining. Values represent means ± S.D. of at least three independent experiments performed in triplicate. Key: (*) p<0.*05* and (**) p<0.*01*, respect to the untreated control cells.

Maslinic acid (2α,3β)-2,3-Dihydroxyolean-12-en-28-oic ac, MA) is a natural compound that belongs to the pentacyclic triterpene family and is widely distributed in the plant kingdom. We isolated this compound from the olive-pressing residues of the pomace, using different solvents, as described by Garcia-Granados et al. [34]. The established industrial procedure for the isolation of maslinic acid from olive fruits (Olea europaea) is also described [35]. It is also available, on a large scale, as solid white powder (85% content, 15% other terpenes compounds) or chemically pure maslinic acid (>97%). Chemically, MA is a made of 30 carbon atoms grouped in five cycles that, as a substitute, have seven methyl groups, two hydroxyl groups and one carboxyl group (Fig 1A). Its molecular weight is 472.7 g/mol. The extract used was a white powder comprising 98% MA and 2% oleanolic acid, which is stable when stored at 4°C. It was dissolved before use at 10 mg/mL in 25% DMSO and 75% PBS. A stock solution was frozen and stored at -20°C. Prior to the experiments, this solution was diluted in cell-culture medium (see below). All experiments were conducted at IC_50_ = 40.7 ± 0.4 μg/mL (Fig 1B) or IC_80_ = 56.8 ± 0.1 μg/mL, the values of MA concentrations required for 50% and 80% growth inhibition after 72 h treatment.

### Cell culture

Human colorectal adenocarcinoma cell lines, Caco-2 (ECACC no. 86010202) and HT29 (ECACC no. 91072701), were provided by the cell bank of the University of Granada (Granada, Spain), and cultured in DMEN medium supplemented with 2 mM glutamine, 10% heat-inactivated FCS, 10,000 units/mL penicillin and 10 mg/mL streptomycin. The cell lines were maintained in a humidified atmosphere with 5% CO_2_ at 37°C. Cells were passaged at pre-confluent densities in a solution containing 0.05% trypsin and 0.5 mM ethylenediaminetetraacetic acid (EDTA). Only subconfluent monolayers of cells were used in all experiments.

### Cell proliferation activity assay

The effect of Ma on proliferation in Caco-2 colon-cancer cells was measured using the MTT assay, which is based on the ability of live cells to cleave the tetrazolium ring, thus producing formazan, which absorbs at 570 nm. Caco-2 cells (15 x 10^3^) were grown in a 96-well plate and incubated with MA (0–100 μg/mL). Following 72 h, 100 μL of MTT solution (0.5 mg/mL) was added to each well. Following 2 h incubation, the cells were washed twice with PBS and the formazan was resuspended in 200 μL DMSO. Relative cell viability was measured by absorbance at 550 nm on an ELISA plate reader (Tecan Sunrise MR20-301, TECAN, Austria).

### Flow cytometric analysis

Flow cytometry was used to measure DNA ploidy as well as alterations in cell-cycle profiles characteristic of DNA fragmentation (necrosis) compared to patterned DNA cleavage (apoptosis). In this way, we were able to visualize cell subpopulations with differing DNA contents. We were able to discern the population size, fractions of nuclei in each phase of the cell cycle, and compute DNA ratios for each nucleus subpopulation identified. Caco-2 cells, seeded at a density of 4 × 10^5^ cells/well, were plated onto 6-well plates with 2 mL of medium. After 24 h, the cells were treated with different doses of MA for 72 h. They were then resuspended by trypsinization and washed in PBS. The cells (6 × 10^5^) were fixed in ice-cold ethanol (70%) for 30 min. The total DNA content was stained with 1 mg/mL propidium iodide (PI). The number of cells at each stage of the cell cycle was estimated by fluorescence-associated cell sorting (FACS) and monitored by flow cytometry. The cell cycle was analyzed using Multicycle software. The data were analyzed to determine the percentage of cells at each phase of the cell cycle (G_0_/G_1_, S and G_2_/M).

### Mitochondrial-membrane potential

Changes in the mitochondrial-membrane potential can be examined by monitoring the cell fluorescence after double staining with Rh123 (rhodamine 123) and PI. Rh123 is a membrane-permeable fluorescent cationic dye that is selectively taken up by mitochondria directly proportional to the MMP (mitochondrial membrane permeabilization). In the same way as in the cell cycle assays, 4 × 10^5^ cells/well were plated on 6-well plates with 2 mL of medium and treated with cytotoxic activity compounds for 4 h at IC_50_ and IC_80_ concentrations. After treatment, the medium was removed and a fresh medium with DHR, at a final concentration of 5 μg/mL, was added. After 30 min of incubation, the medium was removed and the cells were washed and resuspended in PBS with 5μg/mL of PI. The intensity of fluorescence from Rh123 and PI was determined using a FACScan flow cytometer (Coulter Corporation, Hialeah, FL, USA), using excitation and emission wavelengths of 500 and 536 nm, respectively.

### Caspase-8 protease activity

Caspase-8 activity was measured using a colorimetric assay (caspase-8 colorimetric assay kit, Abnova, Tapei, Taiwan) based on the hydrolysis of the peptide substrate Acetyl-Ile-Glu-Thr-Asp-p-nitroaniline (Ac-IETD-pNA) by caspase-8, resulting in the release of the p-nitroaniline. Briefly, 2.7 x 10^6^ Caco-2 cells/well were cultured in 100mm plates and were treated for 72 h with MA at its IC_50_ and IC_80_ concentrations. Similarly, to compare the proposed mechanism with that found in HT29 cells, 1.2 x 10^6^ HT29 cells/well were cultured in the same manner. Cells were lysed with lysis buffer for 10 min on ice, and cell lysates were diluted with the reaction buffer containing 0.2 mM of caspase-8 substrate, and incubated at 37°C for 2 h. The p-nitroaniline concentration was determined by the absorbance measured at 405 nm using an ELISA reader. Fold-increase in caspase-8 activity was determined by comparing with the untreated control cells.

### Cytochrome-c extracts

Caco-2 cells and HT29 cells, under the conditions described in the previous section, were cultured in 100 mm plates and treated for 4 h with MA at its IC_50_ and IC_80_ concentrations. Subsequently, the cells were washed in PBS and resuspended in lysis buffer (20 mM HEPES, pH 7.5, 10 mM KCl, 1.5 mM MgCl2, 1 mM EDTA, 1 mM EGTA, 250 mM sucrose,1 mM dithiothreitol, 0.1 mM phenyl methyl sulfonyl fluoride, 1 μg/mL pepstatin A, 2 μg/mL leupeptin, and 10 μg/mL aprotinine). The samples were centrifuged at 25,000 x g for 30 min at 4°C. The supernatants were further centrifuged at 25,000 x g for 30 min at 4°C and stored at -80°C for cytochrome-c analysis.

### Western blotting

Caco-2 cells (2.7 x 10^6^) and HT29 cells (1.2 x 10^6^) were treated with MA at IC_50_ and IC_80_ concentrations for 4 h, following which they were washed twice with PBS and resuspended in lysis buffer (20 mM Tris/acetate, pH 7.5, 270 mM sucrose, 1 mM EDTA, 1 mM EGTA, 1% Triton X-100, 1 mM orthovanadate, 1 mM sodium glycerophosphate, 5mM sodium fluoride, 1 mM sodium pyrophosphate, 5 mM β-mercaptoethanol, 1 mM bezamidine, 35 μg/ml PMSF, 5 μg/ml leupeptine). The samples were homogenized ultrasonically and incubated on ice for 20 min before being centrifuged at 12,000 x g for 15 min. The supernatants were assayed for protein concentration using a BCA assay kit (Pierce Biotechnology, Rockford, USA).For western-blot analyzes, 25–50 μg proteins were loaded onto 15% SDS-polyacrylamide gel and transferred to a polyvinylidene difluoride membrane (Bio-Rad Laboratories, Richmond, California, USA). The membranes were blocked by incubation in TBS buffer containing 0.1% Tween-20 and 5% dried milk for 1 h at room temperature and washed 3 times with TBS buffer containing 0.1% Tween-20. The membranes were then blotted overnight at 4°C with primary antibodies: rabbit polyclonal anti-caspase-3 and anti-caspase-9 (1/1000 dilution), rabbit polyclonal anti-Bax (1/500 dilution), rabbit polyclonal anti-Bid (1/3000 dilution).To determine caspase-8 and cytochrome-c, membranes were blotted for 1 h at 25°C with a mouse monoclonal primary antibody anti-caspase-8 and anti-cytochrome-c (1/3000). All blots were developed by ECL Western Blotting Detection Kit Reagent (Amersham Biosciencies, Freiburg, Germany) and detected using an LAS-3000 imaging system (Fuji Photo Film Europe, TK Tilburg, The Netherlands).

### Statistics

Statistical analyzes were performed with the GraphPad Prism 5.0 software. All quantitative data were summarized as the means ± standard deviation (SD). For each assay Student`s t test was used for statistical comparison with the untreated control cells. A limit of *p* ≤ 0.05 was considered to be statistically significant. Key: *p* < 0.05 (*), *p* ≤ 0.01 (**) and *p* ≤ 0.001 (***). All data shown here are representative of at least three independent experiments performed in triplicate.

## Results

### Maslinic acid inhibits growth in Caco-2 colon adenocarcinoma cells

Inhibition of Caco-2 colon-cancer cell growth caused by MA was calculated using the MTT assay. Tumor cells were treated with increasing concentrations of MA (0 to 100 μg/mL), their viability determined by formazan dye uptake and expressed as a percentage of untreated control cell proliferation (Fig 1B). The results showed that treatment with MA resulted in a dose-dependent decrease in cell viability after 72 h incubation. The concentration of MA required for 50% growth inhibition (IC_50_) was 40.7 ± 0.4 μg/mL and that for 80% growth inhibition (IC_80_) was 56.8 μg/mL. No correlation was found between cell sensitivity to MA and p53 status i.e. Caco-2 cells do not express p53 but their sensitivity to MA was similar to that of HT29 cells, which do express p53 [36].

### Effect of maslinic acid on the cell cycle of Caco-2 colon-cancer cells

The cell cycle machinery represents an alternative target for the identification of novel biomarkers for cancer detection and prognostication, providing target validation for cell cycle-directed therapies. In the light of the inhibition of cell growth caused by MA, we investigated its effects upon cell-cycle distribution in Caco-2 colon-cancer cells. The distribution of cells in different cell-cycle phases was analyzed by the incorporation of PI after 72 h incubation with MA at IC_50_ and IC_80_ (Fig 1C, top). DNA distribution analysis showed a dose-dependent increase in G_0_/G_1_ phase time, which reached 13% at IC_50_ and 11% at IC_80_ with a significant decrease in the percentage of cells in the S phase (between a 56% - 70%), compared to untreated control cells (Fig 1C, bottom). These results suggest that, in addition to being cytotoxic, MA produces cell-cycle arrest, thus contributing to the inhibition of cell growth. Cell-cycle arrest could also be related to an induction of differentiation by MA in this cell type.

### Maslinic acid induces apoptosis by triggering the activation of caspases-8 and -3

**Fig 2:**
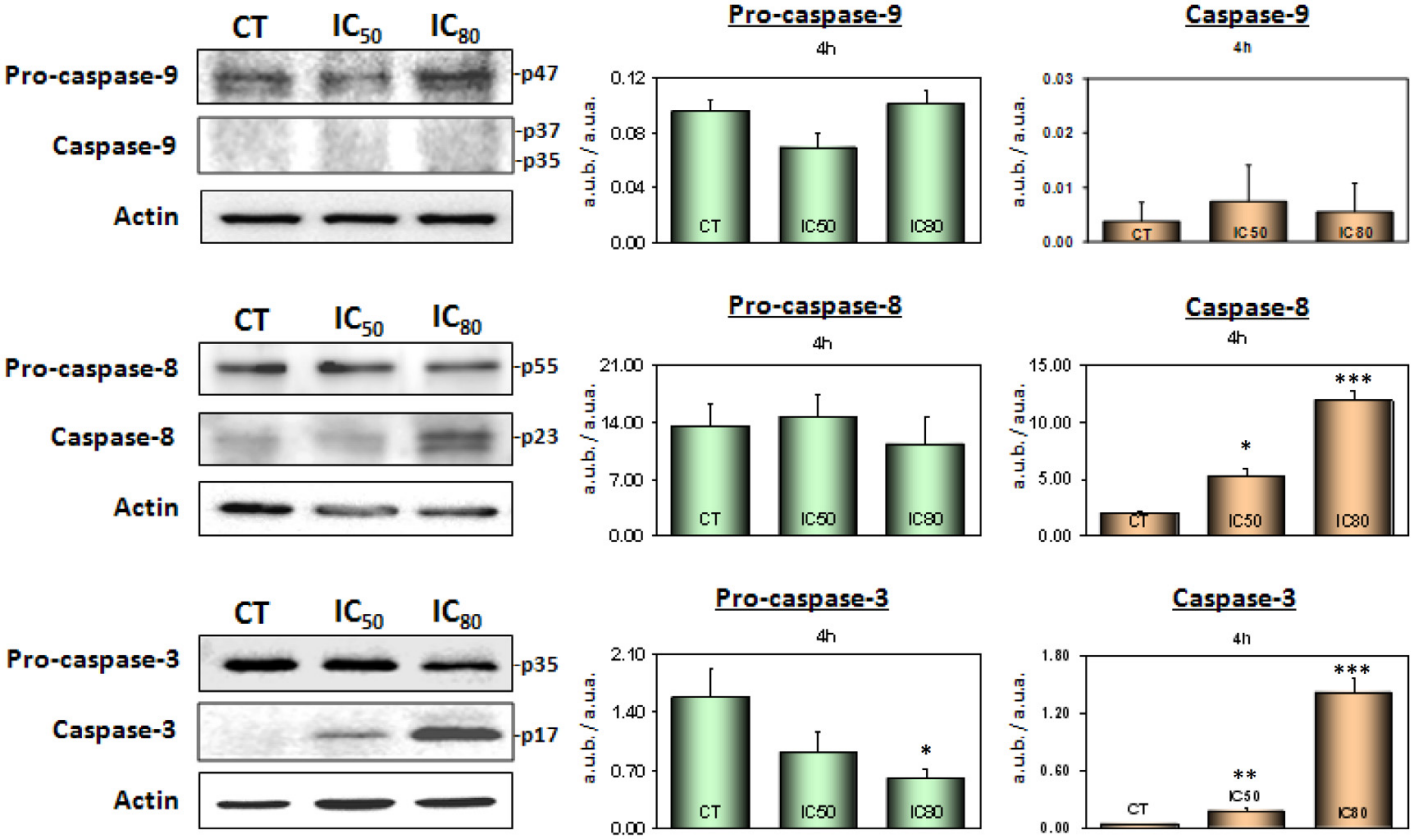
Top: western blotting of the levels of pro-caspase-9 and caspase-9; centre: pro-caspase-8 and caspase-8; and bottom: pro-caspase-3 and caspase-3 proteins. Caco-2 cells were treated with MA at IC_50_ and IC_80_ concentrations for 4 h. The levels of protein expression are expressed as arbitrary intensity units of each band compared to arbitrary intensity units of actin. The values represent means ± S.D. of at least three independent experiments performed in triplicate. Key: (*) p<0.*05*, (**) p<0.*01* and (***) p<0.*001*, with respect to the untreated control cells.

Caspase cascade activation is one of the most important processes in the induction of cell death by apoptosis. Our experiments showed a significant increase in apoptotic cell death in the Caco-2 cell line after 72 h of treatment. Therefore, we studied the rate of caspase-8 and caspase-3 expression from the beginning of treatment with MA (Fig 2). Our results showed evidence of caspase-8 and caspase-3 activation after only 4 h of treatment. These results would indicate the probable triggering of the extrinsic apoptotic pathway in Caco-2 cells in response to treatment with MA, which is contrary to the results we observed previously in HT29 cells, in which apoptosis was clearly shown to be induced via the intrinsic mitochondrial pathway [28].

Following 4 h treatment with MA at both IC_50_ and IC_80_ concentrations, apoptosis in Caco-2 cell cultures was seen to occur via a caspase-8-dependent mechanism. Under these conditions, no changes were found in pro- and caspase-9 levels (Fig 2 top). However, the increase found in the initial caspase-8 levels was 2-fold at IC_50_ and 6-fold at IC_80_ compared to the untreated control cells (Fig 2 middle). In addition, caspase-3 was also clearly activated, indicating that the apoptotic process had been set in motion. Caspase-3 levels at IC_50_ increased by 4-fold, but at IC_80_ this level increased dramatically by 37-fold. These increases in caspase-3 levels were accompanied by a marked cleavage of pro-caspase-3. There was a 45% decrease in pro-caspase-3 at IC_50_ and 65% at IC_80_ after 4 h incubation (Fig 2 bottom).

**Fig 3:**
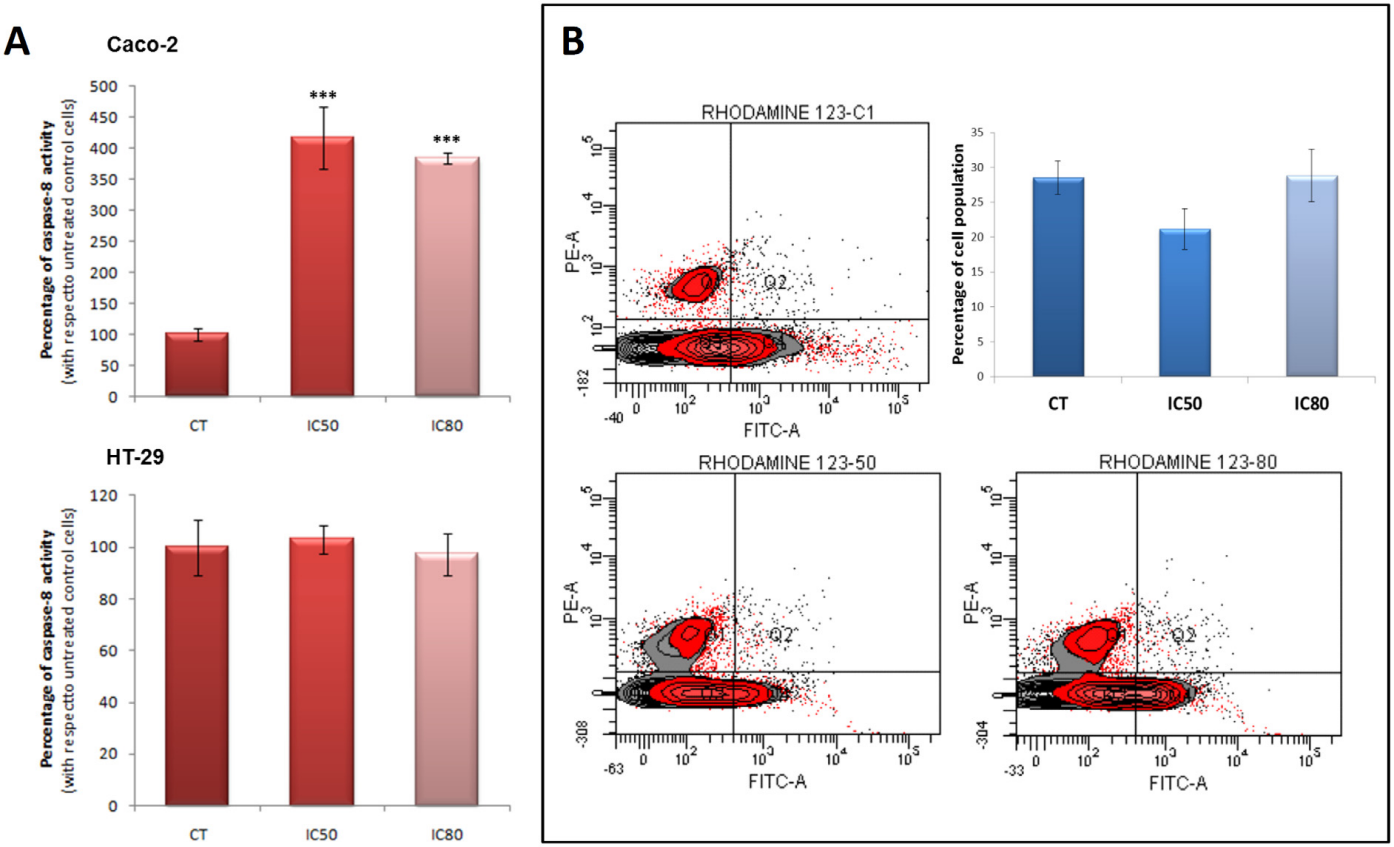
**(Panel A)** Maslinic acid induced apoptosis through activation of caspase-8 in Caco-2 cells (Top), but not in HT29 cells (Bottom). Caco-2 and HT29 cells were treated for 4 h with their corresponding IC_50_ or IC_80_ concentrations. Values represent means ± S.D. of four experiments performed in triplicate. Key (***) P<0.*001*, with respect to the untreated control cells. **(Panel B)** Diagrams of rhodamine 123/propidium iodide flow-cytometry. The right quadrants of each diagram (Q2 and Q4) represent positive cells stained with Rh123, the left quadrants (Q1 and Q3) represent negative cells stained with Rh123. Top-right, percentage of Rh123 positive cell population in control cells (CT), IC_50_ and IC_80_ concentrations, after 4 h of treatment. The values represent means ± SD. of four independent experiments performed in triplicate. Not significant differences were found.

In order to confirm that caspase-8 activation is a part of this mechanism, we determined its activity in Caco-2and HT29cells treated with MA at short time (4 h) and with IC_50_ and IC_80_ concentrations, using colorimetric assay based on the hydrolytic rupture of the peptide Ac-IETD-pNA. As show in Fig 3A, MA caused an important increase in the caspase-8 activity at both concentrations assayed in Caco-2 cells and did not have any effect on this enzyme in HT29 cells.

### Maslinic acid did not trigger the intrinsic mitochondrial apoptotic pathway: Bax, Bid, t-Bid and cytochrome-c

**Fig 4:**
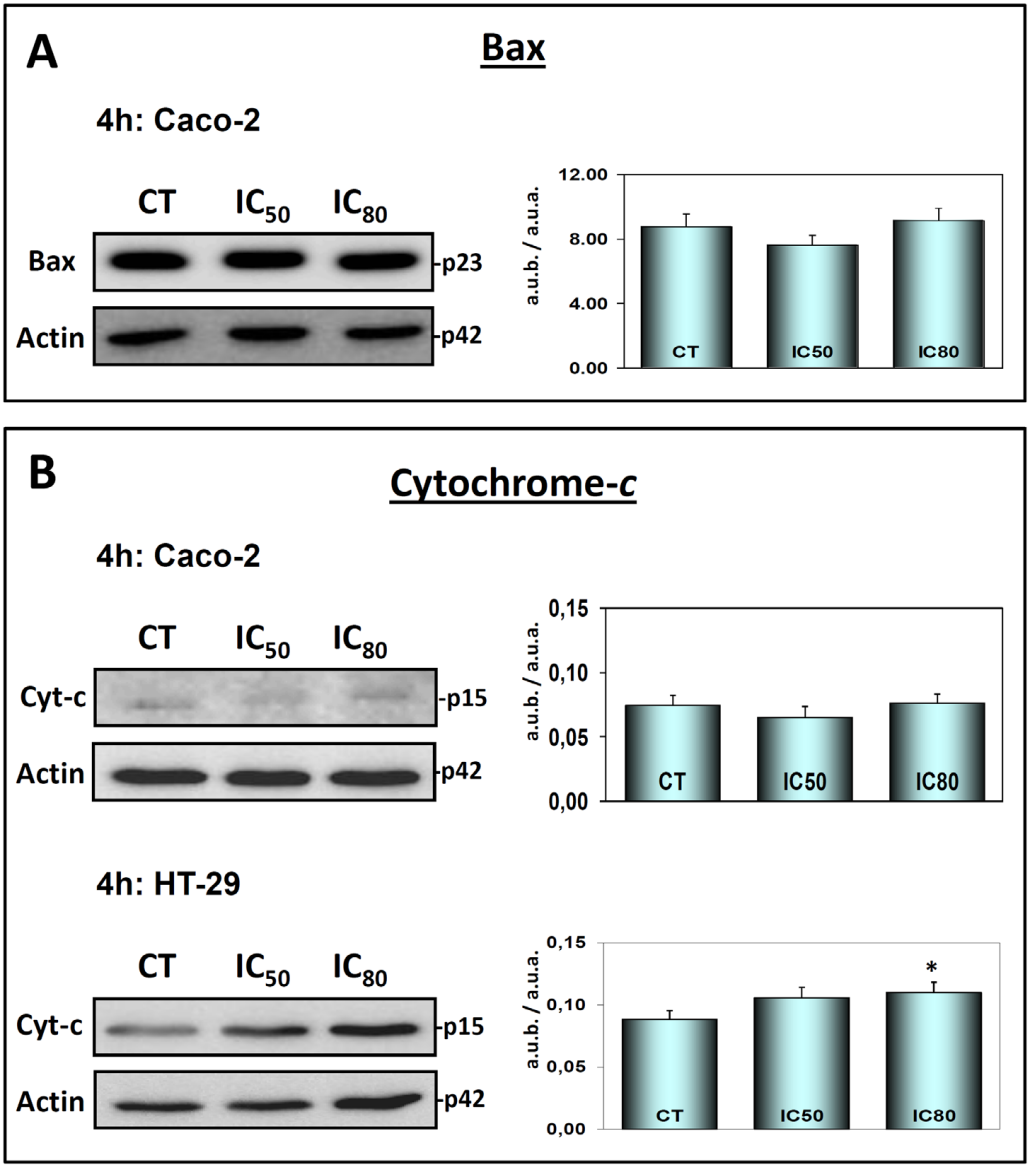
**(Panel A)** Western blotting to determine Bax levels. Caco-2 cells were treated with MA at IC_50_ and IC_80_ concentrations for 4 h. **(Panel B)** Western blotting to determine cytosolic *cytochrome-c* release levels in Caco-2 cells (Top) and HT29 cells (Bottom). Caco-2 and HT29 cells were treated with MA at IC_50_ and IC_80_ concentrations for 4h. The levels of protein expression are expressed as arbitrary intensity units of each band compared to arbitrary intensity units of actin. The values represent means ± SD. of at least three independent experiments performed in triplicate. Key: (*) p<0.*05*, respect to the untreated cells.

To further verify our hypothesis that MA triggers the extrinsic apoptotic pathway in Caco-2 cells, and compare this with the molecular mechanism characterized by us in HT29 cells in a previous study [28], we went on to determine whether MA was capable of triggering the mitochondrial pathway in any way. To this end, we analyzed the expression levels of caspase-9 (Fig 2 top) and the corresponding levels of Bax protein (Fig 4A), which are both essential to the activation of the intrinsic apoptotic pathway. As is well known, Bax is a pro-apoptotic member of the Bcl-2 protein group and induces mitochondrial disruption and the release of cytochrome-c, thus activating caspase-9 followed by Apaf-1 factor, which then turns on the intrinsic caspase cascade.

**Fig 5:**
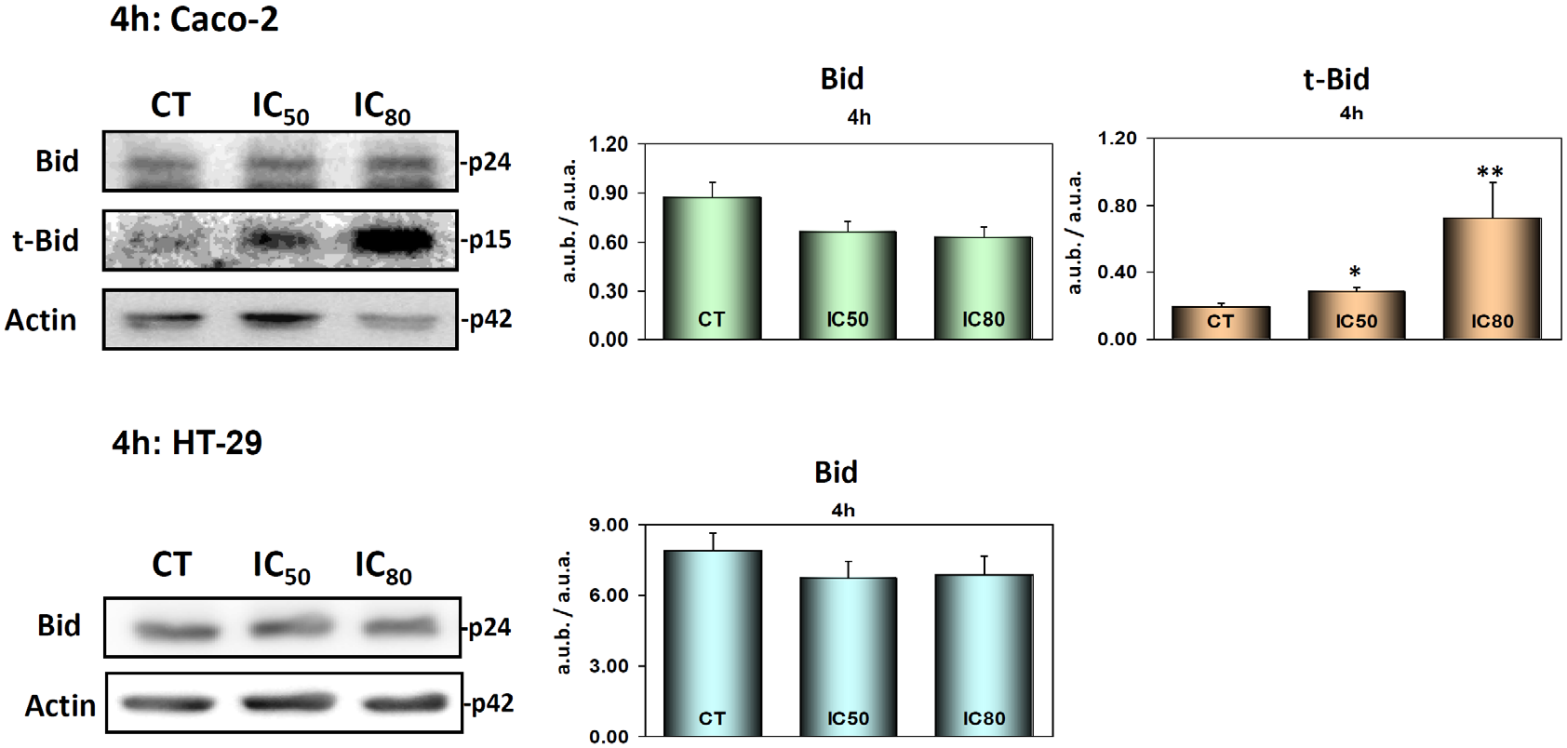
Western blotting to determine Bid and t-Bid levels in Caco-2 cells (Top) and HT29 cells (Bottom). Caco-2 and HT29 cells were treated with MA at IC_50_ and IC_80_ concentrations for 4 h. The levels of protein expression are expressed as arbitrary intensity units of each band compared to arbitrary intensity units of actin. MA produced clear effects on this protein in Caco-2 cells. However, this effect was not observable in HT29 cells. The values represent means ± SD. of at least three independent experiments performed in triplicate. Key: (*) p<0.*05* and (**) p<0.*01*, with respect to the untreated cells.

Furthermore, we examined Bid activation in Caco-2 and HT29 cells (Fig 5) after 4 h of treatment. We observed a decrease of Bid accompanied by a clear increase in the generation of t-Bid (Bid truncate) in response to MA in Caco-2 cells, which was probably cleaved by activated caspase-8. t-Bid is a small active fragment that is generated from the Bid protein and for detection by western blot method, it is necessary to have significant amounts of Bid cleavage. We did not observe this in the HT-29 cells in response to maslinic acid treatment (S1A Fig), although it has been observed at longer incubation times [29].

Our results show that caspase-3 was clearly activated at 4 h incubation at IC_50_ and IC_80_, indicating that the apoptotic process had already been activated, although there was no evidence of either caspase-9 activation or pro-apoptotic Bax levels; t-Bid appears visibly activated, in response to caspase-8. Taken together, these results clearly indicate that MA initially triggers the extrinsic caspase cascade in the Caco-2 cell line via the activation of caspase-8, which in turn activates caspase-3 without activating Bax or caspase-9. This rules-out any induction, over the short term, of the intrinsic apoptotic pathway.

To verify these differences in apoptosis activation mechanisms in Caco-2 and HT29 cells, we examined the cytochrome-c levels in both cell lines at 4 h of treatment (Fig 4B) with MA. The release of cytochrome-c was detected in HT29 cells but not Caco-2 cells, although at low levels. These results suggest that MA has not caused mitochondrial disruption in the activation of apoptosis in Caco-2 cells, confirming the extrinsic apoptotic activation as proposed in the present article. In addition, to confirm that the mitochondrial apoptotic pathway is not involved, changes in mitochondrial membrane permeabilization (MMP) were analyzed by flow-cytometry after staining with Rh123 and PI at 4 h of treatment at IC_50_ and IC_80_ concentrations. The Rh123 results obtained were not significantly different at both MA concentrations (Fig 3B).

## Discussion

The apoptotic process frequently does not function in tumor cells. The generic pathways known to be involved in these processes are the intrinsic mitochondrial-mediated pathway and the extrinsic pathway, which is linked to caspase-8 activation via apoptotic receptors such as FAS, TNFR and TRAIL. Maslinic acid (MA) is a newly discovered, natural, pentacyclic triterpene isolated as the main triterpenoid compound from olive-skin pomace and formerly used as a functional food for animal growth [8,10,15]. In addition, the pentacyclic triterpene family has been shown to have a wide range of potent antitumoral properties and has been reported to trigger both the main apoptotic pathways [9,20,22–25,28,29,33]. In this study, we show the antitumoral effects of MA against the Caco-2 cell line at a very short time.

In a previous study, we described how MA triggered the intrinsic apoptotic process in HT29 colon-cancer cells, mediated by the participation of the Bcl-2 protein group, mitochondrial membrane disturbance, cytochrome-c release and the activation of caspases -9 and -3. MA also induced differentiation in this cell line before the activation of the apoptotic process [28,33]. Contrary to this, the results described in this present report show that, in Caco-2, cells MA triggers the extrinsic apoptotic pathway. Furthermore, a lower cell cycle arrest may indicate a lower differentiation effect in this cell line compared to that in HT29 cells [33]. These results suggest activation of different apoptotic mechanisms in each cell type. Such chemotherapeutic agents, capable of triggering both apoptotic processes, are very interesting from a therapeutic point view.

Firstly, MA caused a significant dose-dependent decrease in cell proliferation: the concentration required for 50% growth inhibition (IC_50_) was 40.7 ± 0.4 μg/mL, and for 80% grow inhibition (IC_80_) was 56.8 μg/mL. These relatively high concentrations could indicate the low toxicity shown by this type of compound. At the doses studied, MA induced a considerable apoptotic effect, as shown by our apoptotic protein expression analysis. Furthermore, MA produced cell-cycle arrest in the G_0_/G_1_ phase (as observed by FACS analysis with propidium iodide stain), which may inhibit cell-growth. The percentage of cell arrest was around 15% at both IC_50_ and IC_80_. This increase during phase G_0_/G_1_ was accompanied by a concomitant decrease in the number of cells in division-phase S. MA produced a significant reduction during phase S, and thereby, contributing to the inhibition of cell growth. Cell-cycle arrest could also be due to induction of differentiation by MA in this cell type.

To determine the activation or inhibition of the proteins involved in the apoptotic mechanism, we assayed apoptotic protein expression after short incubation times and found that after 4 h all the apoptotic proteins were activated. Firstly, we examined caspase-3 expression and found that it was fully activated just a few hours into treatment, indicating the complete induction of the apoptotic process. These results are contrary to our previous results with HT29 cells, in which the complete activation of caspase-3 did not occur until after 72 h of treatment [29]. This may be due to different apoptotic mechanism found in Caco-2 cells compared to HT29 cells, in which apoptosis occurred via the intrinsic pathway [28,29,33].

After determining that executor caspase-3 was activated after only 4 h of incubation, we went on to examine other proteins related to the induction of apoptosis at this time. In contrast to the results found in HT29 cells, the initiator caspase-8 was clearly activated after 4 h treatment, whereas caspase-9 and ROS generation were not observed. This initial activation of caspase-8, the principal signal for the induction of the extrinsic apoptotic pathway, together with the fact that caspase-9 was not activated, indicates that in Caco-2 cells MA induces extrinsic apoptotic activity. This is different to its effect on HT29 cells and other types of cell such as astrocytoma cancer cells, in which it induces intrinsic apoptotic activity [37]. This point was verified by determination of cytosolic cytochrome-c release that was negative for Caco-2 cells.

To verify these results, we then examined the expression levels of the apoptotic factor Bax, a protein also involved in the induction of mitochondrial apoptosis. Bax belongs to the Bcl-2 pro-apoptotic protein group and is involved in the activation of the mitochondrial apoptotic response. Our results showed no Bax activation in the Caco-2 cells in response to MA treatment, which confirms that it triggers the classic extrinsic apoptotic route in these cancer cells (S1B Fig). Although we found the clear activation of Bid protein to t-Bid, probably with the purpose to activate a secondary apoptotic response to initial apoptotic signals mediated by caspase-8-dependent Bid [38,39].

The intrinsic and extrinsic apoptotic pathways not are independent; both can be active crosswise and could include proteins that are activated in both routes. Maslinic acid induces the direct activation of the extrinsic apoptotic route in Caco-2 cells, possibly through membrane receptor. It has been described, for example, that the activation of TNF receptors can induce apoptosis in both ways, depending on the cell line assayed [40,41]. The activation of this type of receptors has been described for other pentacyclic triterpenes, such as the CDDO, an oleanolic acid derivative [42,43], as well as in response to maslinic acid [44,45]. Depending on the number of receptors in these cell lines or a deficiency in the activated molecular route, could lead to the activation of one mechanism or another.

The induction of both apoptosis mechanisms has been widely described in response to different pentacyclic triterpenoids [18,29]. When a cell receives an apoptotic signal, it has to decide between continuing to live or undergo apoptosis. In HT29 cells, maslinic acid induces a clear differentiation effect, mediated by MAP kinase pathway, and the apoptosis process is delayed compared to Caco-2. In Caco-2 cells, the initial apoptotic signal precedes other cell process, through direct activation of caspase-8.This may be related to the fact that Caco-2 cells are double negative for p53 protein (since p53 protein is involved in the differentiation process).

Activation of both the intrinsic and extrinsic routes by other triterpene compounds has been previously described. For example, CDDO and its derivates CDDO-Me and CDDO-Im have been reported to activate the extrinsic apoptotic pathway directly via caspase-8 and caspase-3 [46] in osteosarcoma cells by a mechanism that acts independently of cytochrome-c [47]. Betulinic acid was shown to activate caspase-8, which in turn cleaves caspase-3 [19]. Alternatively, the activation of the intrinsic apoptotic pathway mediated by the cleavage of Bid independently of caspase-8 has also been described [48]. The cleavage of caspases-9, -3, and -8, followed by the activation of Bid and release of cytochrome c, are frequently described phenomena in antitumoral activity induced by triterpenes such as amooranin-AMR [23], CDDO [20,49] and ursolic acid [50].

**Fig 6:**
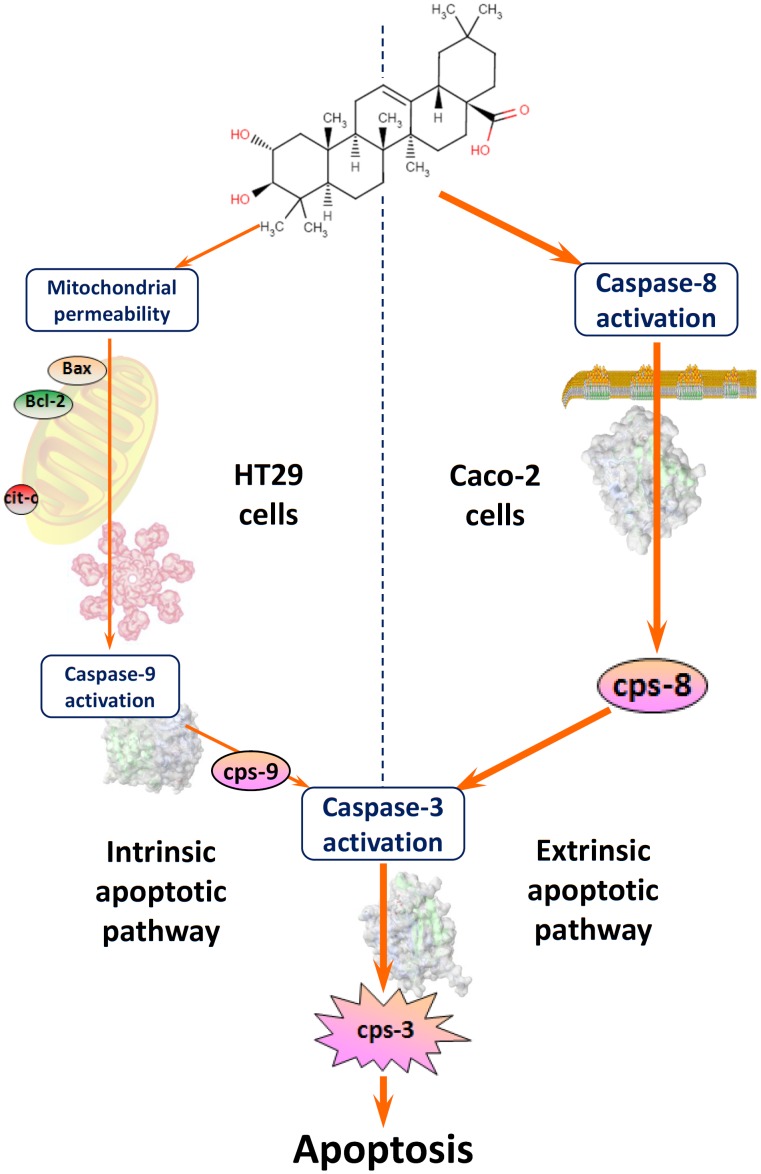
Schematic representation of the different mechanisms proposed for the induction of apoptosis by MA in colon-cancer Caco-2 cells (right) and HT29 cells (left). MA is able to activate both intrinsic and extrinsic apoptotic mechanisms according to the type of cell involved. *Abbreviations*: Cps, caspase; cit c, cytochrome-c.

Based on our results, we propose That MA triggers the extrinsic mechanism for the apoptotis, in the short term, on Caco-2 colon-cancer cells as opposed to the intrinsic mechanism in HT29 colon-cancer cells (Fig 6). To summarize, MA, a novel natural triterpene deriving from olive skins (*Olea europaea* L.), is capable of inducing apoptosis via both the intrinsic and extrinsic apoptotic pathways, depending upon the type of cancer involved. We have shown elsewhere that it triggers the intrinsic mitochondrial-mediated apoptotic pathway in HT29 colon-cancer cells, and in this paper that it triggers the extrinsic caspase-8-mediated apoptotic pathway in Caco-2 colon-cancer cells.

Compounds with the capacity of activating, both, the extrinsic and intrinsic apoptotic routes in adenocarcinoma cells, whether they express protein p53 or not, are very interesting from a pharmacological point of view in that they may lead to a more efficient response. Our results point to the possibility of developing maslinic MA and related drugs for use as chemotherapeutic or chemopreventative agents in tumoural therapy and suggest that it may be an effective compound in the therapy of colon cancer.

## Supporting Information

S1 Fig **(A) Left:** Western bloting of the levels of Bid (p24) and t-Bid (p15) in Caco-2 cells. **Right:** Western bloting of the levels of Bid (p24) in HT29 cells. Note that not levels of t-Bid (p15) were detected in this cell line. Cells were treated with maslinic acid (MA) at IC50 and IC80 concentrations for 4h. **(B) Left:** Western bloting of levels of Bax (p23) in Caco-2 cells. Note that not changes in Bax levels were observed in this cell line. **Right:** Western bloting of levels of Bax (p23) in HT29 cells. Cells were treated with maslinic acid (MA) at IC50 and IC80 concentrations for 4h.
(PDF)
