# Supplementary material for: Maslinic Acid, a Natural Triterpene, Induces a Death Receptor-Mediated Apoptotic Mechanism in Caco-2 p53-Deficient Colon Adenocarcinoma Cells
Source: PLoS One. 2016 Jan 11;11(1):e0146178. doi: 10.1371/journal.pone.0146178 (PMC4709006; doi:10.1371/journal.pone.0146178)
Supplement: S1 Fig — (A) Left: Western bloting of the levels of Bid (p24) and t-Bid (p15) in Caco-2 cells. Right: Western bloting of the levels of Bid (p24) in HT29 cells. Note that not levels of t-Bid (p15) were detected in this cell line. Cells were treated with maslinic acid (MA) at IC50 and IC80 concentrations for 4h. (B) Left: Western bloting of levels of Bax (p23) in Caco-2 cells. Note that not changes in Bax levels were observed in this cell line. Right: Western bloting of levels of Bax (p23) in HT29 cells. Cells were treated with maslinic acid (MA) at IC50 and IC80 concentrations for 4h. (PDF) [file pone.0146178.s001.pdf]

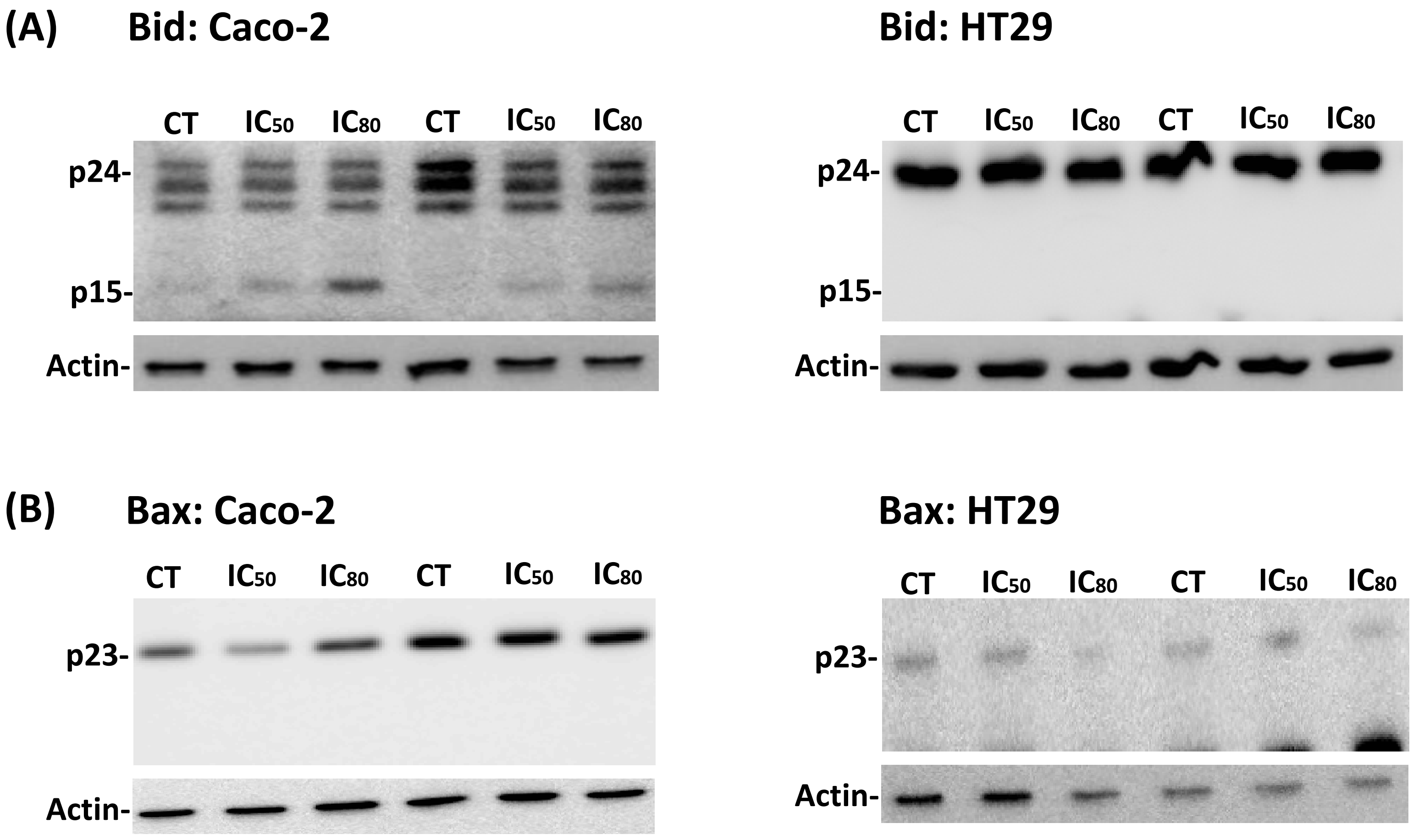

S1 Figure. Supplementary figure 1. (A) Left: Western bloting of the levels of Bid (p24) and t-Bid (p15) in Caco-2 cells. **Right:** Western bloting of the levels of Bid (p24) in HT29 cells. Note that not levels of t-Bid (p15) were detected in this cell line. Cells were treated with maslinic acid (MA) at IC_{50} and IC_{80} concentrations for 4h. **(B) Left:** Western bloting of levels of Bax (p23) in Caco-2 cells. Note that not changes in Bax levels were observed in this cell line. **Right:** Western bloting of levels of Bax (p23) in HT29 cells. Cells were treated with maslinic acid (MA) at IC_{50} and IC_{80} concentrations for 4h.
